# Self-reported Diabetes Mellitus and Risk of Mortality from All Causes, Cardiovascular Disease, and Cancer in Takayama: A Population-based Prospective Cohort Study in Japan

**DOI:** 10.2188/jea.JE2008004

**Published:** 2008-10-01

**Authors:** Shino Oba, Chisato Nagata, Kozue Nakamura, Naoyoshi Takatsuka, Hiroyuki Shimizu

**Affiliations:** 1 Department of Prevention for Lifestyle-related Diseases, Gifu University Graduate School of Medicine, Gifu, Japan; 2 Department of Epidemiology and Preventive Medicine, Gifu University Graduate School of Medicine, Gifu, Japan; 3 Sakihai Institute, Gifu, Japan

**Keywords:** Diabetes mellitus, Mortality, Cardiovascular disease, Cancer, Cohort study

## Abstract

**Background:**

Diabetes mellitus has been reported to be a major risk factor for cardiovascular disease (CVD), and higher risk of CVD among women than that among men has been observed in many studies. Further, the association of diabetes with increasing risk of cancer has also been reported. Well-designed studies conducted among men and women in the general Japanese population remain scarce.

**Methods:**

Our cohort consisted of 13355 men and 15724 women residing in Takayama, Japan, in 1992. At the baseline, the subjects reported diabetes in a questionnaire. Any deaths occurring in the cohort until 1999 were noted by using data from the Office of the National Vital Statistics. The risk of mortality was separately assessed for men and women by using a Cox proportional hazard model after adjusting for age; smoking status; body mass index (BMI); physical activity; years of education; history of hypertension; and intake of total energy, vegetables, fat, and alcohol.

**Results:**

Diabetes significantly increased the risk of mortality from all causes [hazard ratio (HR): 1.35, 95% confidence interval (CI): 1.11-1.64] and from coronary heart disease (CHD) (HR: 2.96, 95% CI: 1.59-5.50) among men, and that from all causes (HR: 1.74, 95% CI: 1.34-2.26) and cancer (HR: 1.88, 95% CI: 1.16-3.05) among women. Diabetes was not significantly associated with mortality from CHD among women.

**Conclusion:**

The findings suggest that diabetes increases the risk of mortality from CVD among men and that from cancer among women. The absence of increased risk of mortality from CHD among women may suggest a particular pattern in the Japanese population.

## INTRODUCTION

Diabetes mellitus is a major risk factor of cardiovascular disease (CVD), and epidemiological studies from different areas have reported that people with diabetes are at higher risk of mortality from CVD and from all causes.^1^^-^^4^ Japan was ranked fifth among the World Health Organization member states in terms of the estimated number of cases of diabetes, and the increased risk of mortality among people with diabetes imposes a significant health burden on the nation. A previous study reported that the relative effect of diabetes on the risks of mortality from CVD and all causes among the Asian population did not differ from those among the Caucasian population.^5^ The same study also reported that the CVD risks associated with diabetes were similar in men and women. However, detailed information regarding each cohort from Japan that contributed to the study has not yet been published and was therefore unavailable. Another study in Japan indicated that the increase in the risk of heart disease among diabetic patients was slightly higher in men than in women, although the study was not based on an underlying observational epidemiological study, and comparison was made between the data of diabetic patients and population statistics.^6^ One prospective study in Japan reported that the presence of diabetes increased the risk of CVD, and another study conducted on atomic bomb survivors reported an association between HbA1c and mortality from CVD.^7^^,^^8^ In both these studies, the combined risks for men and women were calculated, and the number of participants included was rather limited. Another study has reported an increase in the risk of CVD in diabetic patients,^9^ although the participants of this study were not selected from the general population but consisted of patients. Thus, a study on a large cohort of men and women selected from the general Japanese population in order to assess the relationship between diabetes and the risk of mortality from CVD and all causes is desired.

It has also been reported that diabetes is associated with an increased risk of cancer. Moreover, studies conducted in Japan and those conducted abroad have reported a relationship of the risk of cancer or specific cancer in several sites with diabetes and with the HbA1c and fasting plasma glucose levels.^8^^,^^10^^-^^14^ Cancer is the leading cause of death in Japan, and mortality from cancer exceeds that from CVD in both men and women.^15^^,^^16^ The risk of cancer mortality in diabetic patients is likely to have an impact on the nation’s public health.

The aim of the current study is to conduct a prospective cohort study to assess the association between diabetes and mortality from CVD, cancer, and all causes. We examined the male-female difference in the risks of mortality associated with diabetes. Our cohort was community-based and was selected from the general Japanese population, and the risks were assessed after considering the risk factors for CVD and cancer.

## METHODS

### Study Participants

The data were obtained from the Takayama study in Japan. The details of the Takayama study have been described elsewhere.^17^^-^^19^ In brief, the study population consisted of men and women who were residents of Takayama City and were 35 years or older in 1992. At the baseline, a self-administered questionnaire was administered to 36990 residents, and a 92.0% response rate was obtained. Of the participants who responded to the questionnaire, those who did not complete more than 45% of it (n = 595, 1.7%) and those who gave unreliable or inconsistent responses (n = 1871, 5.5%) were excluded from the cohort. The final fixed cohort consisted of 31552 subjects, including 14427 men and 17125 women. Physician diagnoses of diabetes and other major diseases, including hypertension, were reported in the questionnaire in response to the question “Have you ever been told by a physician that you have following diseases?” The participants answered this question for each listed disease. Those who reported a history of cancer, myocardial infarction, angina, or stroke were excluded from the cohort. The final cohort for the current study consisted of 29079 subjects, including 13355 men and 15724 women.

Information regarding the baseline characteristics of the study cohort, such as age; height; weight; cigarette smoking; use of medication, including aspirin; and length of education in years, was reported in the questionnaire. Women’s health issues, including menopausal status and use of hormone replacement therapy, were also asked in the questionnaire. In addition, a semi-quantitative, validated, food-frequency questionnaire (FFQ) that quantified 169 food items was administered.^17^ From the FFQ data, the total daily calorie intake and the intake of each nutrient and food item were estimated according to the Japanese Standard Tables of Food Composition, 5^th^ edition, published by the Science and Technology Agency of Japan. Detailed information on the FFQ, including its validity and reproducibility, has been previously described.^17^ The amount of regular physical activity was estimated from the validated questionnaire and expressed in terms of metabolic equivalents per week.^20^^,^^21^

### Ascertainment of Mortality

Deaths in the cohort that occurred between September 1992 and December 1999 were recorded. After obtaining permission from the Ministry of Internal Affairs and Communication to review the data regarding deaths, each cause of death was confirmed using the data from the Office of the National Vital Statistics. The major endpoint of this study was mortality from all causes, CVD, cancer, and causes other than cancer and CVD. We further analyzed mortality from several diseases that were frequently observed in the cohort. The Statistics and Information Department of the Japanese Ministry of Health and Welfare listed all the causes of deaths, which were coded according to the International Classification of Diseases, 10^th^ Revision (ICD-10). Deaths from cancer were classified as codes C00 through C97, and deaths from CVD, as codes I00 through I99 and Q25 through Q28.

### Data Analysis

The age-adjusted mortality rates per 10000 person-years classified according to the status of diabetes were separately calculated for men and women by standardization to the rates among subjects of the Takayama study by sex and by 10-year age category. We compared the characteristics of the participants with and without diabetes by using *t* tests for continuous variables and chi-square tests for categorical variables. The intake of each food and nutrient was logarithmically transformed for statistical testing in order to approximately normalize its distribution. To assess the magnitude of the association of diabetes with mortality from each cause, a Cox proportional hazard model was applied to estimate hazard ratios (HRs) with 95% confidence intervals (CIs). To track the subjects who moved out of the study area, we referred to the city residential registers, and these subjects were counted as censored subjects. Age was included in the model for adjustment since it is a potent risk factor for diabetes, CVD, and cancer. We also considered a multivariate model with adjustments for other factors that were associated with diabetes: age; smoking status; body mass index (BMI); physical activity; years of education; history of hypertension; total energy intake; and intake of vegetables, fat, and alcohol. The intake of each food and nutrient in the model was adjusted for the total energy intake by using the regression analysis proposed by Willett.^22^ All statistical analyses were performed using SAS (SAS Institute Inc., Gary, NC).

## RESULTS

At the baseline, 5.9% males and 2.7% females reported that they had diabetes. Table 1 summarizes the baseline characteristics of the participants by the diabetes status in both men and women. The subjects with diabetes were significantly older, and their BMI was slightly but significantly higher than that of non-diabetic subjects. The subjects with diabetes were less physically active, more frequently reported a history of hypertension, and their caloric intake was significantly lower than the subjects without diabetes.

**Table 1.** Baseline characteristics of 13355 men and 15724 women by diabetes status in Takayama, Japan, 1992-1999

	Men					Women				
	
	Without Diabetes		With Diabetes		Two-sided	Without Diabetes		With Diabetes		Two-sided
	n = 12561		n = 794		*P* value^†^	n = 15301		n = 423		*P* value^†^
	Mean ( ± standard deviation)					Mean ( ± standard deviation)				
	
Age (y)	53.7	(12.1)	58.5	(11.0)	<0.01	54.9	(13.0)	63.1	(11.8)	<0.01
Body mass index (kg/m^2^)	22.5	(2.8)	23.0	(2.8)	<0.01	22.0	(2.9)	22.4	(3.3)	<0.01
Height (cm)	164.8	(6.8)	163.7	(6.8)	<0.01	152.1	(6.4)	150.1	(6.1)	<0.01
Physical activity (MET/week)	27.3	(41.7)	23.4	(38.3)	0.01	18.9	(29.8)	14.4	(23.1)	<0.01
Total energy intake (kcal/d)	2610	(868)	2504	(872)	<0.01	2115	(778)	1877	(708)	<0.01
Total vegetable intake (g/d)	370.0	(258.6)	408.1	(287.3)	<0.01	393.4	(264.2)	431.9	(288.7)	0.01
Total fat intake (g/d)	61.2	(28.6)	60.7	(27.8)	0.64	55.5	(26.6)	49.3	(25.0)	<0.01
Total alcohol intake (g/d)	42.1	(41.4)	39.5	(43.2)	<0.01	7.8	(16.9)	4.3	(11.8)	<0.01
	

	No. (%)					No. (%)				
	
Currently married	11380	(90.6)	713	(89.8)	0.46	11380	(74.4)	251	(59.3)	<0.01
Education 12 years or more	5358	(42.7)	299	(37.7)	0.01	5151	(33.7)	85	(20.1)	<0.01
Cigarette smoking status^*^										
Never smoker	2051	(16.3)	127	(16.0)	<0.01	11328	(74.0)	296	(70.0)	0.04
Current smoker	6757	(53.8)	375	(47.2)		1800	(11.8)	52	(12.3)	
Former smoker	3394	(27.0)	265	(33.4)		600	(3.9)	14	(3.3)	

History of hypertension	2279	(18.1)	246	(31.0)	<0.01	2605	(17.0)	124	(29.3)	<0.01
Aspirin use within the past 6 months	508	(4.0)	39	(4.9)	0.23	1,043	(6.8)	19	(4.5)	0.06
Post-menopausal women						8680	(56.7)	351	(83.0)	<0.01
Current hormone replacement therapy use						239	(1.6)	10	(2.4)	0.19

* Do not add up to 100% because of missing data

† *t* test for continuous variables and chi-square test for categorical variables

During the follow up, 1163 deaths occurred in men over 91036.7 person-years, and 899 deaths occurred in women over 110123.1 person-years. Standardized mortality rates by the diabetes status and the HRs of mortality among men are shown in Table 2. The risks of mortality from all causes and from coronary heart disease (CHD) were significantly higher among diabetic men than among non-diabetic men. Further analysis of the disease-specific mortality by using multivariate adjustment showed an increased risk of mortality from liver cancer (HR: 4.30, 95% CI: 1.98-9.38) among diabetic men as compared with that among non-diabetic men.

**Table 2.** Age-adjusted mortality from cardiovascular disease, cancers, and all causes of death and the hazard ratios (HRs) of the mortalities among diabetic and non-diabetic men in the Takayama study

Cause of death	ICD-10 Code	Men without diabetes		Men with diabetes		Age-adjusted HR	95% CI		Multivariate^†^ HR	95% CI	
	
		Death (n)	Mortality rate per 10000 person-year*	Death (n)	Mortality rate per 10000 person-year*						
All deaths		1050	124.83	113	165.32	1.26	1.04	1.53	1.35	1.11	1.64
Cancer	C00-C97	363	43.24	37	53.62	1.21	0.87	1.70	1.33	0.94	1.87
Cardiovascular disease	I00-I99	267	31.73	41	60.20	1.79	1.29	2.49	1.82	1.30	2.53
Coronary heart disease	I20-I25	45	5.36	13	18.37	3.05	1.66	5.61	2.96	1.59	5.50
Stroke	I60-I64, I67, I69, Q25-Q28	120	14.27	17	23.91	1.64	0.99	2.73	1.65	0.99	2.76
Deaths not from cancer or cardiovascular disease		420	49.86	35	51.50	0.97	0.69	1.37	1.05	0.74	1.49

* Age standardized to that of the male participants of the Takayama study

† Adjusted for age; smoking status; BMI; physical activity; length of education in years; history of hypertension; total energy intake; and intake of vegetables, fat, and alcohol

ICD-10: International Classification of Diseases, 10^th^ revision, CI: confidence interval

The standardized mortality rates by the status of diabetes and the HRs of mortality among women are shown in Table 3. The risks of mortality from all causes, cancer, and causes other than cancer and CVD were significantly higher among diabetic women than among non-diabetic women. The risks of mortality from CHD and stroke, and the total CVD risk did not differ between diabetic and non-diabetic women. Further analysis of the mortality from several diseases by using multivariate adjustment revealed a higher risk of mortality from colorectal cancer (HR: 4.30, 95% CI: 1.78-10.41) among diabetic women than among non-diabetic women. We repeated the analysis after excluding women who reported the current use of hormone replacement therapy, and the results were essentially the same.

**Table 3.** Age-adjusted mortality from cardiovascular disease, cancers, and all causes of death and the hazard ratios (HRs) of the mortalities among diabetic and non-diabetic women in the Takayama study

Cause of death	ICD-10 Code	Women without diabetes		Women with diabetes		Age-adjusted HR	95% CI		Multivariate^†^ HR	95% CI	
	
		Death (n)	Mortality rate per 10000 person-year*	Death (n)	Mortality rate per 10000 person-year*						
All deaths		836	79.26	63	137.65	1.71	1.32	2.21	1.74	1.34	2.26
Cancer	C00-C97	235	22.19	18	38.58	1.92	1.19	3.10	1.88	1.16	3.05
Cardiovascular disease	I00-I99	309	29.36	18	35.59	1.31	0.81	2.10	1.36	0.84	2.20
Coronary heart disease	I20-I25	46	4.37	2	3.85	0.91	0.22	3.73	0.49	0.07	3.57
Stroke	I60-I64, I67, I69, Q25-Q28	127	12.06	5	10.34	0.88	0.36	2.15	0.88	0.36	2.16
Deaths not from cancer or cardiovascular disease		292	27.70	27	63.48	2.09	1.41	3.10	2.09	1.39	3.14

* Age standardized to that of the female participants of the Takayama study

† Adjusted for age; smoking status; BMI; physical activity; length of education in years; history of hypertension; total energy intake; and intake of vegetables, fat and alcohol

ICD-10: International Classification of Diseases, 10^th^ revision, CI: confidence interval

## DISCUSSION

The current study suggests that self-reported diabetes increases the risk of mortality from all causes in men and women, from CVD in men, and from cancer in women. The results partially contradict those of previous studies conducted mainly in Western countries, which repeatedly reported that diabetic women have a higher risk of CVD and that they lose their advantages over men regarding CVD.^4^^,^^23^^-^^30^ It was reported in a review of previously conducted epidemiological studies that the age-adjusted mortality rates for CHD were 2 to 3 times higher among diabetic men and 3 to 7 times higher among diabetic women in population-based studies.^29^ However, since 1996, 4 meta-analyses were conducted on the topic of the higher risk of CHD among women than among men, and the results were rather contradictory: Three studies concluded that compared with men, women with diabetes were at increased risk of mortality from CHD, and 1 study found no difference between men and women.^3^^,^^4^^,^^31^^,^^32^

A previous study in Japan compared data from patients with diabetes and population statistics and showed that the risk of heart disease among diabetic women did not exceed that among diabetic men. The ratio of the observed number of deaths from heart disease among diabetic patients to the number expected on the basis of population statistics was 1.93 (*P* < 0.01) in men and 1.58 (not significant) in women.^6^ A study that assessed the association between random blood glucose levels and the risk of ischemic stroke also did not show a distinct difference in the risk between diabetic men and women; the relative risk was 1.8 (95% CI 1.0-3.2) for men and 2.2 (95% CI 1.2-4.0) for women.^33^ These 2 studies were not included in the above meta-analyses; they might not meet the inclusion criteria because of their study design or because the participants were not selected from the general population. Other prospective cohort studies in Japan did not assess the relationship between diabetes and the risk of mortality for men and women separately; instead, sex was adjusted in the model, presumably because of the limited number of participants or because the studies were not originally concerned about the sex differences in the magnitude of risk.^7^^-^^9^

The previously reported higher risk of CVD among women with diabetes relative to that among men with diabetes might be due to obesity, since obesity has been observed to be more prevalent among diabetic women than diabetic men in several studies conducted in the US.^30^^,^^34^^,^^35^ In our cohort, the mean BMI among women with diabetes (22.0 kg/m^2^) was nearly equal to the mean BMI among women without diabetes (22.4 kg/m^2^), but the difference between the two was nevertheless statistically significant. We considered the possibility that women with lower BMIs were more likely to report diabetes. Consistent with the findings of the current study, the findings of previous studies showed that the BMIs of people with diabetes were similar to those of the general population in Japan.^36^ The average BMI was 23.1 kg/m^2^ among male and female participants with diabetes in the Japan Diabetes Complications Study,^37^ while the average BMI among a similar age group in the general Japanese population ranged between 22.90-23.70 kg/m^2^.^38^ The smoking status may also have influenced the association between diabetes and mortality from CVD among women since it had been previously reported that cigarette smoking increased the risk of CVD mortality among women with diabetes.^39^ However, only about 15% of women were current or former smokers in our study. Our stratified analysis by smoking status failed to show any differences of the risks between the stratum with regard to the mortality from CVD among diabetic women compared to that among non-diabetic women (data not shown). Nonetheless, we cannot eliminate the possibility that as compared to men, women in the current study had less severe diabetes, and that this was responsible for the smaller risk of CVD observed among women. Information on the severity of diabetes was unavailable in the current study.

In the current study, diabetic women had increased risk of mortality from cancer. This may be partly attributable to the increased risk of colon and colorectal cancer observed among diabetic women. In contrast, an association between diabetes and mortality from cancer was not found among men. The results of a previous prospective cohort study that assessed the risk of mortality from colon/colorectal cancer were inconsistent in terms of variation by sex.^11^^,^^40^^,^^41^

The risk of mortality from causes other than CVD and cancer was higher among diabetic women than among non-diabetic women. Of the causes of mortality other than cancer and CVD, diabetes was the most frequently observed among diabetic women. A total of 12 diabetic women died as a result of diabetes (ICD-10 codes: E10-E14), and the age-standardized mortality rate from all causes other than cancer and CVD was 27.27 per 10000 person-years, which was relatively higher than the equivalent rate among diabetic men, 14.06 per 10000 person-years. Further detailed information on mortality was not available; however, mortality as a result of diabetes may be caused by acute complications, and we speculate that this might have overtaken the mortality from CVD.

The current study has several advantages. The study was conducted in a community-based cohort selected from the general Japanese population, and the participation rate was relatively high. Mortality within the cohort was prospectively followed up, and deaths from all causes were confirmed using the data from the Ministry. Potential multiple confounders of the association between the status of diabetes and mortality were adjusted for the analysis.

Nevertheless, the current study has several limitations. The diagnosis of diabetes was reported in a questionnaire, and the validity and reliability of the report was undetermined. Fortunately, the positive predictive value for self-reported diabetes among Japanese subjects in a previous study was high (82%), and substantial agreement was found between the diabetic patients identified using questionnaires and those identified using confirmed medical records.^42^ Nevertheless, it is possible that a certain proportion of men and women who had diabetes did not report it in the current study. A study conducted by the Japanese Ministry of Health, Labour and Welfare estimated that the prevalence of diabetes among Japanese people who were 50 years or older was more than 14.2% in men and more than 7.1% in women in 1997. These values are considerably higher than those estimated at the baseline of the current study for both men and women, although in the study by the Health Ministry, the estimations were made using the hemoglobin A1c test, and this study was conducted 5 years after the initiation of the current study.^43^ Such underreporting could introduce bias in the estimation of the association between diabetes and mortality. Despite the limitations of self-reported diabetes, as in the current study, several previous large-scale epidemiological studies among the general population used self-reported diabetes to assess its association with the risk of cancer or other conditions.^12^^,^^42^ Further, some other studies assessed this association and did not use the oral glucose test or medical record review to validate self-reported diabetes.^11^^,^^44^^-^^46^ Such misclassification, caused by the underestimation of the true prevalence of diabetes, would bias the analysis of the association toward the null when true association exists, and thereby attenuate the association. Furthermore, information on diabetes was only available at the baseline, and diabetes that may have developed during the follow-up period was not considered. Data on hyperlipidemia, a traditional risk factor of CVD, were not available in our study. In a recent report from the Asia Pacific Cohort Studies Collaboration,^47^ the mean total cholesterol level was 5.34 mmol/L in people with diabetes and 5.11 mmol/L in people without diabetes, and these levels are relatively similar. The risk of CHD increased both for persons with diabetes and for persons without diabetes; an approximately 2-fold increase was observed when the highest fourth of the total cholesterol (approximately 6.3 mmol/L) was compared to the lowest fourth (approximately 4.5 mmol/L).^47^ Considering the 3-fold increase in the risk of mortality from CHD in men with diabetes observed in the current study, the positive association would not be negated even after taking the effect of total cholesterol into account. Moreover, the size of the cohort may not have been sufficiently large to assess the risk of mortality, especially considering the mortality from some diseases with relatively low mortality rates.

Despite the limitations, the current study provides valuable information regarding the risk of mortality among diabetic men and women. For both men and women, an increased risk of mortality from all causes was suggested. Among men with self-reported diabetes, an increased risk of death from CVD was observed, and among women with self-reported diabetes, an increased risk of death from cancer was observed. The observed variation in the risk by sex was not fully explained in the current study, but with further investigation, a distinct pattern of the risk of mortality among people with diabetes in the Japanese population may emerge.
